# A comparison of childbirth self-efficacy, fear of childbirth, and labor pain intensity between primiparas and multiparas during the latent phase of labor: a cross-sectional study

**DOI:** 10.1186/s12884-024-06571-3

**Published:** 2024-05-31

**Authors:** Yue Huang, Yuehua Zhong, Qiaozhu Chen, Jun Zhou, Bailing Fu, Yongfang Deng, Xianfang Tu, Yingfang Wu

**Affiliations:** Department of Obstetrics, Guangzhou Women and Children Medical Center, Guangzhou Medical University, No.9 Jinsui Road, Tianhe District, Guangzhou, 510623 China

**Keywords:** Primipara, Multipara, Childbirth self-efficacy, Fear of childbirth, Labor pain, A cross-sectional study

## Abstract

**Background:**

Childbirth is a long-lasting physiological stress. As one of the main stressors, labor pain exists throughout the whole process. Childbirth self-efficacy is the confidence, or belief that they can manage pain during childbirth. This sense of self-efficacy determines how pregnant women deal with labor pain and enables them to regulate their behavior and actively deal with childbirth. However, the difference in pain sensitivity between single births (primiparas) and multiple births (multiparas) has rarely been investigated.

**Objectives:**

This study is aimed at investigating self-efficacy, fear of childbirth, labor pain of primiparas and multiparas and exploring factors related to the perceived labor pain intensity of pregnant women.

**Design:**

Prospective cross-sectional study.

**Setting(s):**

Labour and delivery in a large academic specialized hospital in Guangzhou, China.

**Participants:**

A total of 347 women, (182 primiparas and 165 multiparas) were enrolled in the data analysis. Pain was assessed before cervical dilatation (cervical dilatation ≤ 3 cm for the first delivery and ≤ 2 cm for the second delivery).

**Method:**

The general information of participants was obtained by questionnaire and obstetrical records of the subjects were obtained from the electronic medical records extracted from the electronic medical record system (EMRS). Childbirth self-efficacy, fear of childbirth (FOC) and labor pain were compared between primiparas and multiparas. Paired t-test, chi-square test, Mann–Whitney test, univariate and multivariate regression analysis were used to analyze labor pain between the two groups and investigate factors related perceived labor pain intensity.

**Results:**

The total scores related to fear of childbirth, fetal health, self-control, and labor pain injury of multiparas were notably reduced compared with primiparas (all *P* < 0.05). The perceived labor pain intensity and duration of the first stage of labor was reduced in the multipara group compared with the primipara group. The childbirth control sense of the multipara was better than that of the primipara. The perceived labor pain intensity was negatively correlated with advanced age (age ≥ 35 years), self-efficacy score, family support, and education (all *P* < 0.05). In contrast, the perceived labor pain intensity was positively correlated with tension, severe fear of childbirth, and anxiety (*P* < 0.05). Self-efficacy, gravidity, delivery cognition, and fear of childbirth were independent risk factors for the perceived labor pain intensity in the latent period (all *P* < 0.05).

**Conclusions:**

Fear of childbirth is a predictor of perceived labor pain intensity. The extent of labor pain (minimum and maximum) can be predicted by the level of fear the expectant mother has. During the latent phase of labor, self-efficacy, fear of childbirth and labor pain are different between primiparas and multiparas.

## Introduction

Childbirth is a natural physiological process of human reproduction and labor pain is a process that almost all women can experience [1]. Labor pain exists in the whole process of childbirth and is a paroxysmal physiological pain [2]. Labor pain is caused by the paroxysmal contraction of uterine muscles and the dilation and compression of the birth canal during childbirth [3]. Many methods are available to relieve labor pain. Currently, the most common method is epidural labor analgesia. Epidural labor analgesia blocks the transmission of pain nerves through epidural drugs to maintain maternal and infant safety and relieve labor pain during delivery of pregnant women [4, 5]. Fear of childbirth (FOC) is the fear of unknown events, such as labor pain and adverse events. Due to the lack of experience related to childbirth, primiparas are more likely to generate fear, anxiety, and other bad feelings more than multiparas. A large body of evidence has supported that severe fear of childbirth might increase the risk of preterm childbirth, dystocia, elective cesarean section, and emergency cesarean Sects. [6–8]. The expected labor pain during the latent phase could reduce the strong stress of the parturient and help the parturient to maintain her physical fitness and cooperate to complete the delivery. The accurate expectation of pain can eliminate the bad emotions such as tension and anxiety caused by the coming pain and calmly face childbirth [9, 10].

Childbirth self-efficacy refers to a woman’s confidence or belief that she can manage pain during delivery, such as deep breathing, relaxation, and distraction to reduce pain [11]. Self-efficacy is an essential concept in social cognitive theory, which is people’s belief in the ability to successfully achieve behavioral goals or deal with difficult situations [12]. Childbirth self-efficacy is the confidence, or belief that they can manage pain during childbirth. This sense of self-efficacy determines how pregnant women deal with labor pain and enable them to regulate their behavior and actively deal with childbirth [13]. When uterine contraction emerges, a good sense of childbirth control can help pregnant women control pain, establish a positive psychological state and enhance their enthusiasm, participation, and intelligence in medical decision-making [14, 15].

Due to the three-child policy, there are more and more multiparas in China [16]. Since the fear of childbirth, self-efficacy, childbirth control, and labor pain of primiparas and multiparas are different [12], it is necessary to elucidate the associated factors. The adjustment of fertility policy also allows more high-risk multiparas. When multipara becomes pregnant again, their FOC will increase due to physical and mental changes [17]. On the other hand, due to the lack of knowledge and experience related to delivery, primiparas are also prone to destructive emotions such as fear and anxiety. Emerging evidence suggests that severe FOC might reduce pregnant women’s self-efficacy in delivery. Published studies have shown that labor pain is not a simple reflection of the physiological process of delivery but the result of the interaction of various physiological and psychological factors [18]. In addition, several studies have shown that childbirth self-efficacy is associated with delivery pain [19]. However, a systematic understanding of the association among delivery fear, childbirth self-efficacy and perceived labor pain intensity in primiparas and multiparas remains insufficient. Therefore, we carried out a prospective cross-sectional study to investigate self-efficacy, fear, and labor pain of both primiparas and multiparas to explore factors related to perceived labor pain intensity. We aim to investigate: (1) the childbirth control sense between the multiparas and primiparas; (2) the comparation of the perceived labor pain intensity and duration between the multiparas and primiparas in the first stage; (3) the relationship among the perceived labor pain intensity and self-efficacy score, family support, and education, and (4) the independent risk factors for the perceived labor pain intensity in the latent period.

## Methods

### Study design and participants

This cross-sectional study was conducted in a large academic hospital from April to October 2020. In recent years, the number of births in our hospital has been about 30,000 per year, ranking first in Guangdong Province. Women who underwent spontaneous or induced labor were enrolled by convenient sampling immediately at the beginning of the childbirth process. The inclusion criteria were women with singleton pregnancy (gestational age 36 weeks) between the ages of 20 and 40, without uterine scar, without mental disorders history, without serious pregnancy complications such as heart disease, uncontrolled hypertension, and gestational diabetes, without contraindications for epidural analgesia, able to read Chinese. Exclusion criteria: persons who did not complete the questionnaire. Two nurses in the obstetric ward were trained as investigators. Obstetric nurses include midwives, midwives can provide prenatal, intrapartum and postnatal care services, especially can be responsible for delivering babies, obstetric nurses mainly provide prenatal and postnatal services in China. Laboring women who agreed to participate in this study were invited to rate their pain and fill out the required questionnaires to report their feelings about childbirth.

### Sociodemographic and obstetrical characteristics

Demographic and obstetrical characteristics were retrieved from the structured electronic medical records system (EMRS), including maternal age, body mass index (BMI), gravidity, gestational week, labor duration, pregnancy outcomes, and whether received Epidural analgesia. In addition, a questionnaire was used to survey education level, only child or not, satisfaction with spouse support, participation in prenatal training, desired delivery mode, and their biggest concerns in the delivery.

### Sample size

The determination of sample size is critical to ensure adequate power of detecting the anticipated effects. In this study, the sample size was calculated using the formula by comparing two means with a significance level (\(\alpha\)) of 0.05 and a power (\(\beta\)) of 0.80. The expected effect size was 0.5, standard deviation 5.5, and a desired difference in means of 0.2.

Then, we can use the formula for a two-sample t-test with equal variances: \[ n = \left(\frac{\text{Effect Size} \times \text{Standard Deviation}}{\text{Difference in Means}/\sqrt{2}}\right)^2 \].

The calculation of sample size was performed using G*Power software version 3.1.9.2. The assumptions for this calculation were made based on previous literature and expert consultation. It is important to note that a 5% attrition rate was accounted for in the sample size calculation, resulting in a final target sample size of 347 participants.

### Questionnaires

#### Childbirth attitudes questionnaires (CAQ)

According to Lowe’s Childbirth Attitudes Questionnaires (CAQ) [20], Wei Juan modified it and proposed a Chinese a vision [21]. The fear of childbirth scale (FOS) in this study, which consists of 16 items with a rating of 1 ∼ 4 (1-never; 2-mild; 3-moderate; 4-high). The total score is 16 ∼ 64; the higher the score the more serious the fear of childbirth: 16 ∼ 27, no fear of childbirth; 28 ∼ 39, mild fear of childbirth; 40 ∼ 51, moderate fear of childbirth; and 52 ∼ 64, severe fear of childbirth. Cronbach’s alpha of the Chinese Version of Childbirth Attitudes Questionnaire was 0.916, and Cronbach’s alpha of each dimension was 0.678–0.853. The test-retest reliability of the questionnaire was 0.812–0.921, and the content validity index was 0.924 [22].

#### The childbirth self-efficacy scale (CSES)

The childbirth self-efficacy scale (short form of 32-item Chinese childbirth self-efficacy inventory, CBSEI-C32) [23] was used to evaluate the self-efficacy of pregnant women. The scale consists of two parallel subscales: outcome expectation (outcome expectancy subscale, OE-16) and self-efficacy expectation (efficacy expectancy subscale, EE-16). This scale covers 32 items, including two parallel sub-scales (outcome expectation and childbirth self-efficacy scales). Each sub-scale consists of 16 items, each scored according to 10 grades, with a total score of 320. The higher the score, the higher the sense of self-efficacy during childbirth. In this study, Cronbach’s α coefficient of the scale is 0.91, which shows strong reliability.

#### The labor agentry scale (LAS)

Women’s sense of control during childbirth was measured by the Labor Agentry Scale (LAS) [24], which includes 22 items, each with 7 grades. A total score for each item was acquired through inverting the scores of positively worded items (7 = 1,6 = 2, etc.) and then summing the data. The total score range of the scale is 29 ∼ 203. The higher the score, the stronger the individual’s control over the childbirth process and the more positive the mood. Self-Rating Anxiety Scale (SAS) was used to evaluate the content of maternal anxiety; the evaluation scale contains 20 items, with a standard score of 50, mild anxiety of 50 to 59, moderate anxiety of 60 to 69, and severe anxiety of 60 to 69.

Childbirth attitudes questionnaire and the childbirth self-efficacy scale were filled in during labor. The childbirth control scale was filled in 24–48 h after childbirth. The data was collected by trained researchers. The researchers checked that there were no missing items.

#### Numerical rating scale (NRS)

The Numerical Rating Scale (NRS) is a method used to assess and quantify pain intensity. It consists of a scale ranging from 0 to 10, where 0 represents no pain and 10 represents the worst possible pain. Patients are asked to indicate their level of pain by choosing a number on the scale that best corresponds to their experience. The NRS is a commonly used tool in clinical settings for the evaluation and monitoring of pain perception.

### Labor pain assessment and pregnancy outcomes

Pain of labouring women in the latent phase was assessed before cervical dilation ≤ 3 cm for the first childbirth and ≤ 2 cm for the second childbirth [20]. The Numerical Rating Scale (NRS) was used to evaluate the pain of parturient women during the first stage of labor: 0, painlessness; 10, severe pain. A 10-point scale is introduced: level 0 (painless), 0; level I (mild pain), 1 ∼ 3; II (moderate pain), 4 ∼ 7; III (severe pain), 8 ∼ 10. The mode of delivery, duration of the first stage of labor and pregnancy outcomes were recorded.

### Ethical and legal considerations

The protocol was approved by our hospital’s Institutional Ethics Review Committee (NO. 2021.113A01). This study was carried out according to the ethical standards of the Declaration of Helsinki of the World Medical Association. All subjects signed a written and informed consent form, and written informed consent was obtained in the latent phase from all participants. Data used in this study were anonymous, and no identifiable personal data of the patients were available for analysis.

### Statistical analysis

Data processing was performed with SPSS 22.00 software (IBM Company, Armonk, NY, USA). Descriptive analysis was carried out to assess the demographic and clinical characteristics of the subjects. Continuous variables were expressed as mean ± SD, and categorical variables were described as n, percentages. Paired t-test or Mann–Whitney test was used for continuous variables and chi-square tests for categorical variables to evaluate the difference between the primipara and multipara groups. The mixed-effects model was applied to determine the connotation of sociodemographic and clinical factors with perceived labor pain intensity. Perceived labor pain intensity referred to the subjective experience and rating of pain during labor as reported by the laboring individual. This measure involved the individual’s own assessment and description of the intensity of pain they were feeling during the labor process. Perceived labor pain intensity may vary greatly among individuals and may be influenced by factors such as the individual’s pain tolerance, emotional state, prior experiences with pain, and the stage of labor. Logistic regression analysis was used to evaluate the relationship between a categorical dependent variable and one or more independent variables. Independent variables that showed a significant association with the dependent variable in univariate logistic regression (*P* ≤ 0.05) were considered for inclusion in the multivariate analysis. Then, the multivariate logistic regression analysis was carried out based on the candidate factors identified by the univariate regression analysis. The independent prognostic factors were determined by the multivariate Cox proportional hazards regression analysis, and the regression coefficient and hazard ratios (HRs) were calculated by the Cox regression model. The odds ratio (OR) was considered as the risk factor evaluation indicator, and OR > 1 was the risk factor of perceived labor pain intensity. *P* < 0.05 was considered statistically significant.

## Results

### Demographic and clinical characteristics

A total of 347 women, including 182 primiparas and 165 multiparas, were included for data analysis. Table 1 shows the demographic and clinical characteristics of the two groups. No statistical difference was observed between the primiparas and multiparas in the demographic and clinical characteristics (all *P* > 0.001).

**Table 1:** Demographic characteristics of primiparas and multiparas

Variables	Primiparas (*n* = 182)	Multiparas (*n* = 165)	χ2	*P*
**Age (years)**			2.941	0.035
≤ 35	178 (97.81%)	136(82.42%)		
> 35	4 (2.19%)	29(17.58%)		
**Education**			4.567	0.022
Junior high school and below	10(5.49%)	18(10.91%)		
High school or technical school	15(8.24%)	26(15.76%)		
College	142(78.02%)	106(64.24%)		
Graduate or above	15(8.25%)	15(9.09%)		
**Epidural analgesia received**			0.734	0.463
Yes	161(88.46%)	116(70.30%)		
No	21(11.54%)	49(29.70%)		
**Pre-pregnancy BMI (kg/m2)**			5.649	0.059
< 20	6(3.30%)	2(1.21%)		
20-24.2	70(38.46%)	48(29.09%)		
> 24.2	106((58.24%)	115(69.70%)		
**Family support**			0.857	0.392
Insufficient support	3(1.65%)	5(3.03%)		
Full support	179(98.35%)	160(96.97%)		
**Participation in prenatal training**			0.304	0.066
Yes	40 (21.98%)	29 (17.58%)		
No	142 (78.02%)	136 (82.42%)		
**Desired delivery mode**			0.223	0.096
Vaginal delivery	118 (64.84%)	105 (63.64%)		
Cesarean section	64 (35.16%)	60 (36.36%)		
**Preterm childbirth**			0.734	0.463
Yes	6(3.30%)	8(4.85%)		
No	176(96.70%)	157(95.15%)		

BMI: body mass index. Data were presented as n (%) and analyzed by chi-square test

### Fear of childbirth of primiparas and multiparas

Among the 347 pregnant women, there were 182 primiparas and 165 multiparas. The fear of childbirth of primiparas and multiparas was demonstrated in Tables 2 and 3. There were no significant differences in the rate of no FOC and mild FOC (*P* > 0.05, Table 2). In addition, the rates of moderate and severe fear of childbirth of multiparas were significantly lesser than those of primiparas (*P* < 0.05, Table 2). The total scores related to fear of childbirth, fetal health, self-control, and labor pain injury of multiparas were notably reduced compared with primiparas (Table 3, *P* < 0.05).

**Table 2:** Fear of childbirth for primiparas and multiparas

Variables	Primiparas (*n* = 182)	Multiparas (*n* = 165)		χ2	*P*
No fear of childbirth	54(29.67%)		68(41.12%)	2.487	0.059
Mild fear of childbirth	77(42.31%)		70(42.42%)	1.587	0.065
Moderate fear of childbirth	47(25.82%)		26(15.75%)	2.048	0.009
Severe fear of childbirth	4(2.20%)		1(0.61%)	3.491	0.018

Data were presented as n, %

**Table 3:** Details about fear of childbirth and childbirth self-efficacy for primiparas and multiparas

Classification	Primiparas (*n* = 182)		Multiparas (*n* = 165)	z		*P*	
The total score related to FOC	33.346 ± 9.182	30.410 ± 8.580		3.066		0.002	
Fetal health	11.819 ± 3.479	10.960 ± 3.464		2.291		0.023	
Self-control	8.813 ± 2.767	7.952 ± 2.498		3.033		0.003	
Labor pain injury	8.358 ± 2.414	8.275 ± 2.639		3.366		0.001	
Medical care	4.440 ± 1.751	4.139 ± 1.448		1.730		0.085	
Childbirth self-efficacy outcome expectation	103.7 ± 31.28	106.9 ± 30.73		2.596		0.3301	
The expectation score of childbirth self-efficacy	105.7 ± 30.11	108.0 ± 29.54		1.058		0.459	
The total score of childbirth self-efficacy	209.4 ± 60.39	215.0 ± 58.84		1.960		0.381	
LAS score	108.5 ± 19.55	115.1 ± 19.09		2.974		0.002	

Data were expressed as ‾x ± s and analyzed by Mann-Whitney test or t text. FOC, fear of childbirth

### The perceived labor pain intensity and duration of the first stage of labor of primiparas and multiparas

The perceived labor pain intensity score was delimited as the highest NRS score recorded during the latent period of the first stage of labor. In the current study, duration of the first stage of labor in the multipara (6.407 ± 4.547) group was significantly lower or shorter than that in the primipara (9.901 ± 4.236) group (*P* < 0.0001). There was no significant difference in the NRS score of the perceived labor pain intensity between the two groups (*P* > 0.05). The perceived labor pain intensity of the first stage of labor of the multiparas (5.036 ± 2.072) was no less than that of primiparas (5.220 ± 1.903). Therefore, there was no significant difference in pain between multiparas and primiparas in the first stage of labor, where uterine contraction was the primary source of pain.

### Childbirth self-efficacy of primiparas and multiparas

Childbirth self-efficacy refers to the confidence or belief that pregnant women can complete pain coping strategies to reduce pain during delivery. Anxiety, education, understanding of the delivery process, lecture participation, and delivery experience might influence pregnant women’s childbirth self-efficacy. As shown in Table 4, the total scores of childbirth self-efficacy in the primipara and multipara groups were 209.4 ± 60.39 and 215.0 ± 58.84, respectively. Furthermore, the scores related to childbirth self-efficacy outcome expectation in primipara and multipara groups were 103.7 ± 31.28 and 106.9 ± 30.73, respectively. The expectation scores of childbirth self-efficacy in primipara and multipara groups were 105.7 ± 30.11 and 108.0 ± 29.54, respectively. There was no significant difference in childbirth self-efficacy between the two groups (*P* > 0.05) (Table 3). The results of this study exposed that parity might not affect the childbirth self-efficacy of pregnant women.

### Childbirth control of primiparas and multiparas

The sense of childbirth control refers to the feelings of the parturient during delivery, including the control of their behavior and uterine contraction. LAS scores were used to evaluate the childbirth control of primiparas and multiparas. Compared with the primipara group, the LAS scores in the multipara group were markedly higher (*P* < 0.05, Table 3), suggesting that the childbirth control sense of the multipara was better than that of the primipara. Due to a lack of delivery experience, primiparas’ delivery fear and psychological burden might increase. At the same time, multiparas possess relatively higher delivery ability and safety perception, can better tolerate labor pain, and control the labor process, so they have a higher sense of delivery control.

### Univariate logistic regression analysis of the factors related to the perceived labor pain intensity

The results of the univariate logistic regression analysis demonstrated that the perceived labor pain intensity was negatively correlated with advanced age (age ≥ 35 years), self-efficacy score, family support, and education (*P* < 0.05), with correlation coefficients of -0.1904, -4.705, -2.8095, -0.1563, and − 0.8876, respectively. In contrast, the perceived labor pain intensity was positively correlated with tension, severe fear of childbirth, and anxiety (*P* < 0.05, correlation coefficient = 0.8793) (Table 4).

**Table 4:** Univariate logistic regression analysis of factors related to the perceived labor pain intensity

Appearances	β	SE	Wald	*P*	Odds ratio (95%CI)
**Age (years)**					
< 35	0.128	0.100	1.600	0.209	0.223(0.076 to 0.755)
≥ 35	-0.190	0.110	2.976	0.006	1.263(1.070 to 1.613)
**Self-efficacy**					
Help childbirth self-efficacy	-4.705	1.599	8.662	0.004	3.619(2.374 to 9.035)
Face childbirth self-efficacy	-2.810	0.477	0.246	0.038	1.138(1.015 to 1.764)
**Gravidity**					
< 2	-0.027	0.654	0.169	0.686	0.049(0.018 to 0.644)
≥ 2	0.991	0.110	0.138	0.907	0.459(0.258 to 0.964)
**Fear of childbirth**					
Severe	1.150	0.579	0.094	0.014	1.045(0.096 to 1.550)
Not severe	0.982	0.163	1.099	0.905	0.513(0.338 to 1.671)
**Childbirth cognition**	0.628	0.069	0.811	0.369	0.160(0.093 to 0.904)
**Family support**	-0.156	0.263	2.571	0.036	1.150(1.088 to 1.914)
**Education**	-0.888	1.075	0.681	0.017	0.653(0.176 to 0.928)
**Tension and anxiety**	0.879	0.686	0.146	0.008	0.873(0.673 to 2.945)

Data were expressed as ‾x ± s and analyzed by univariate analysis. Childbirth cognition was represented as continuous variable. Family support, gravidity, tension and anxiety, fear of childbirth, self-efficacy, and age were represented as binomial classification variables. Education was represented as multi-classification variable

### Multivariate logistic regression analysis of the factors related to the perceived labor pain intensity

The multivariate logistic analysis was carried out with perceived labor pain intensity score as dependent variable, self-efficacy, gravidity, fear of childbirth, and childbirth cognition as latency dependent variables. The results showed that self-efficacy, gravidity, delivery cognition, and fear of childbirth were independent risk factors for the perceived labor pain intensity in the latent period (*P* < 0.05, Table 5).

**Table 5:** Multivariate logistic regression analysis of factors related to the perceived labor pain intensity score

Independent variable	β	SE	*P*	OR	95% CI
Self-efficacy	-0.466	0.148	< 0.0001	3.305	-2.289 to -4.822
Gravidity	0.305	0.183	< 0.0001	2.034	0.955 to 0.978
Severe fear of childbirth	2.268	0.203	< 0.0001	3.450	1.876 to 2.672
Not severe fear of childbirth	2.440	0.431	< 0.0001	3.338	1.230 to 2.787
Childbirth cognition	-0.034	0.006	< 0.0001	4.226	-0.046 to -0.023

Data were expressed as ‾x ± s and analyzed by multivariate logistic regression analysis. Childbirth cognition was represented as continuous variable. Gravidity and fear of childbirth were represented as binomial classification variables. Education was represented as multi-classification variable

## Discussion

In the current study, we investigated FOC, self-efficacy, childbirth control, and labor pain of 182 primiparas and 165 multiparas. We firstly validated that primiparas had a higher level of childbirth fear. Fear of childbirth is a common adverse psychological reaction of pregnant women. There are many reasons to propose that fear generation is closely related to parity. Adams et al. [25] enrolled 2,206 pregnant women in Norway to conduct empirical analysis and research. The results showed that the probability of FOC in primiparas was much higher than that in multiparas. Körükcüet et al. [26] analyzed 660 pregnant women. They found that the total W-DEQ score of primiparas was higher than that of multiparas, and they were likely to be afraid of delivery. The above evidence verifies the association between FOC and parity. Consistent with these earlier findings, this study assessed FOC and parity, and found that primiparas had advanced FOC compared with multiparas. Parity is an essential factor that affects the FOC. Because primiparas experience the process of delivery for the first time, they will have more worry or fear that their behavior will get out of control because of labor pain [27]. Moreover, some multiparas might also have varying degrees of FOC because of their previous lousy childbirth experience. For example, maternal and fetal injuries, severe pain, and medical intervention might cause stress disorder (PTSD) during childbirth, resulting in FOC during the second pregnancy, and a survey was conducted on primiparas and multiparas and found that multiparas who had a history of lateral incision or midwifery were more likely to report FOC [20, 28, 29],. Størksen et al. [30] performed a survey of pregnant women from 2009 to 2011. They observed that 8.6% of multiparas experienced a poor delivery history, so they were more afraid of the delivery process, and 4.8 times more afraid of delivery than other pregnant women. This study used FCS to evaluate the FOC of 182 primiparas and 165 multiparas. It was observed that fear of childbirth, fetal health, self-control, and labor pain injury of multiparas were notably reduced compared with primiparas. There were no significant differences in the rate of no FOC and mild FOC. On the contrary, the rates of moderate and severe FOC of multiparas were significantly lower than those of primiparas. The primiparas showed higher FOC; this might be due to their fear of the unknown process of delivery, and they were more worried about the baby’s safety during delivery.

This study used LAS to measure women’s perceived control during delivery. It was found that the LAS scores in the multipara group were markedly higher compared with the primipara group, suggesting that the childbirth control sense of the multipara was better than the primipara. It has been previously shown that childbirth control is closely related to the cognition of the delivery process [31]. Emerging evidence suggests that improving the cognitive awareness of delivery can improve the maternal sense of self-efficacy and reduce the pressure of pregnancy [32]. Delivery is an intense emotional and physical experience for every parturient. Several studies have indicated multiparas had a higher level of delivery cognition during delivery than primiparas [33]. Consistent with these observations, this study showed that the sense of childbirth control of the multipara is better than that of the primipara, which may be related to their higher delivery ability and safety perception. At the same time, multiparas possess relatively higher delivery ability and safety perception, can better tolerate labor pain, and control the labor process, so they have a higher sense of delivery control [34]. With the progress of midwifery technology, measures such as accompanying delivery, family delivery room, and free posture should be taken to improve the parturient’s sense of delivery control. Therefore, we should improve pregnant women’s correct understanding of delivery and strengthen education on childbirth knowledge.

Previous studies have indicated that childbirth self-efficacy of multiparas is higher than that of primiparas, indicating a correlation between childbirth self-efficacy and childbirth experience [35]. Consistently, in the current study, there was no significant difference in childbirth self-efficacy between primiparas and multiparas. The results of this study indicated that parity might not affect the childbirth self-efficacy of pregnant women. This result may be explained by the fact that the pregnant women involved in our study were from the obstetrical ward of a tertiary maternal and child health hospital, which adopted the evidence-based practice of non-drug analgesia. The evidence-based practice of non-drug analgesia plan markedly enhanced the obstetric nursing service. Furthermore, the pregnant women in our survey have mastered some awareness of childbirth. These parturients, especially all the primiparas participated in delivery-related health education and non-drug analgesia learning before delivery. The nurse provided one-to-one support on non-drug labor analgesia for pregnant women and their families. In addition, the maternal labor pain was assessed regularly, and non-drug analgesia strategies were adjusted according to maternal personal preferences and adaptability. The above professional and personalized support could significantly improve the maternal sense of security and ease the pain of childbirth. These parturients are more confident in natural delivery. They have a significantly higher sense of self-efficacy, which may explain why parity in this study might not affect the childbirth self-efficacy of pregnant women.

The stress response caused by pain significantly increases the risk of childbirth, especially for primiparas, because the delivery process is unknown, generating tension and fear, which is not conducive to successful childbirth [36]. In this study, the univariate analysis demonstrated that the perceived labor pain intensity was negatively correlated with advanced age (aged ≥ 35 years), self-efficacy, family support, and education. In contrast, the perceived labor pain intensity was positively correlated with tension and anxiety. In addition, the results of multivariate logistic regression analysis demonstrated that the fear of childbirth is an independent risk factor for perceived labor pain intensity, while childbirth cognition, good social support and good relationship between the husband and wife are protective (mitigating) factors of active pain. These findings suggest that fear is a crucial factor that causes pain during delivery. At the same time, maternal knowledge about childbirth can reduce maternal anxiety and fear to a certain extent, reducing maternal pain during delivery [37]. Therefore, it is critical to take effective nursing interventions according to perceived labor pain intensity risk factors. Targeted care, such as encouragement, prenatal midwifer-led psycho-educational interventions, health education during childbirth, accompanying support during childbirth, and sedation, can effectively improve the fear of childbirth and encourage women to reduce labor pain. The cognition and experience of delivery affect the psychological and behavioral response of pregnant women, which is closely related to the fear and confidence of delivery [38]. Obstetrical nurses should actively take measures to improve pregnant women’s mastery of delivery knowledge through multi-entry point experience guidance, situational exercises, and on-site guidance. Nurses should provide psychological guidance to pregnant women and give them professional information guidance during pregnancy. Through delivery experience, delivery skills training (guide pregnant women to learn delivery breathing relaxation, progressive muscle relaxation), and other ways to reduce parturients’s fear of delivery and enhance their delivery confidence. The nursing staff instructed the family members to provide more support to parturients, to meet their psychological and spiritual needs as much as possible, to understand, encourage and accompany them, and to inform the family members of the importance of good social support to maternal emotional regulation.

To bookend the study, under China’s fertility policy change, parity is an important factor influencing perceived labor pain intensity. Navigating the novel field of directing women’s psychological labor pain, this study found that the fear of childbirth predicts perceived labor pain intensity. During the latent phase of labor, childbirth control sense, fear of childbirth and labor pain are different between primiparas and multiparas. However, our study showed that childbirth control did not strongly predict perceived labor pain intensity. The role and value of childbirth control in pain management during delivery remain to be further investigated. This study has not observed a significant difference in childbirth self-efficacy between primiparas and multiparas during the latent phase of labor. Further study is required to investigate the role of childbirth self-efficacy in perceived labor pain intensity supervision during delivery. We emphasize that health care staffs evaluate pain support requests in parturients of diverse parities enthusiastically and promptly. Our responsibility is to implement non-drug and pharmacological pain relief interventions for women in childbirth to improve their delivery experience.

Clinically, we should pay attention to the possible fear of delivery among pregnant women, find out the causes of delivery fear, and deal with it pertinently, to reduce its influence on childbirth self-efficacy. We should strengthen prenatal psychological counseling to reduce or eliminate the fear of delivery of pregnant women; use standardized prenatal education to make pregnant women master the skills and knowledge related to delivery, to reduce their fear of the unknown.

This study has some limitations. The patients included in this retrospective study were from a single center. Due to the regional limitation, some patients with incomplete information were excluded. Moreover, the current study was limited by the truth that the variables were retrospectively extracted. The evaluation scales employed in clinical backgrounds was the limited self-report instruments. The deficiency of other assessments about pain intensity might not be as effective as when it is gathered in the prospective study. In addition, although the scale used to evaluate FOC in this study has been verified among pregnant women in China, it has not yet been validated among women giving birth globally. Consequently, it is suggested that the findings on distinctive cultural backgrounds or clinical conditions might be cited with prudence.

In summary, fear of childbirth is an independent risk factor of perceived labor pain intensity. During the latent phase of labor, self-efficacy, fear of childbirth and labor pain of primiparas and multiparas are different. In clinical research, medical staffs should actively make use of relevant support to explore targeted and effective prenatal education models, enhance pregnant women’s sense of self-efficacy and reduce their fear of delivery, to promote maternal and infant health.

## Data Availability

The data that support the findings of this study are available from Guangzhou Women and Children’s Medical Center but restrictions apply to the availability of these data, which were used under license for the current study, and so are not publicly available. Data are however available from the authors upon reasonable request and with permission of Guangzhou Women and Children’s Medical Center.
